# Supplementary material for: Dynalign II: common secondary structure prediction for RNA homologs with domain insertions
Source: Nucleic Acids Res. 2014 Nov 21;42(22):13939–48. doi: 10.1093/nar/gku1172 (PMC4267632; doi:10.1093/nar/gku1172)
Supplement: SUPPLEMENTARY DATA [file supp_gku1172_nar-02021-z-2014-File012.zip › manual/javadoc/comments/InterfaceSourceWindows.html]

A package that holds all internal windows used in the drawing framework.
  
  
The classes include not only the visual windows, but their actions as well.
These actions include both GUI manipulation and back end calculations.
